# The Interplay between Gut Microbiota and Cognitive Functioning in the Healthy Aging Population: A Systematic Review

**DOI:** 10.3390/nu16060852

**Published:** 2024-03-15

**Authors:** Maria Kossowska, Sylwia Olejniczak, Marcelina Karbowiak, Wioletta Mosiej, Dorota Zielińska, Aneta Brzezicka

**Affiliations:** 1 Institute of Psychology, SWPS University, 03-815 Warsaw, Poland; mkossowska1@swps.edu.pl (M.K.); solejniczak1@st.swps.edu.pl (S.O.); 2 Institute of Human Nutrition Sciences, Warsaw University of Life Sciences (SGGW), Nowoursynowska 159c, 02-776 Warsaw, Poland; marcelina_karbowiak@sggw.edu.pl (M.K.); wioletta_mosiej@sggw.edu.pl (W.M.); dorota_zielinska@sggw.edu.pl (D.Z.)

**Keywords:** gut microbiota composition, cognitive functioning, healthy aging, Alzheimer’s disease predictors

## Abstract

Background: The gut microbiota in healthy older individuals typically show a decrease in beneficial bacteria like Bifidobacterium and Lactobacillus, alongside an increase in pro-inflammatory microbes such as Enterobacteriaceae and Clostridia. These changes contrast with younger and middle-aged individuals and appear to correlate with cognitive status. Although there is extensive research on gut microbiota and cognitive functions in cognitively impaired elderly individuals, its impact on cognitively healthy elderly populations has not been extensively studied. Method: A comprehensive literature search was conducted across PubMed, EBSCO, Web of Science, and Scopus databases to identify studies exploring the relationship between gut microbiota composition and cognitive functioning in healthy older adults. During the literature screening process, each record was initially assessed by its title, abstract, and keywords to exclude articles that did not align with the scope of this review. Three authors independently screened and retrieved the records. The inclusion criteria included: (1) publication in peer-reviewed journals; (2) studies involving neurologically, cognitively, and medically healthy populations; (3) participants identified as older adults, defined for this review as individuals aged 45 years and older due to the limited number of records; (4) analysis of gut microbiota; and (5) assessment of cognitive function. Subsequently, full texts were analyzed to determine eligibility. The exclusion criteria encompassed: (1) incorrect publication type; (2) inappropriate sample population; (3) unsuitable study design; (4) absence of one or more inclusion criteria; and (5) studies based on animal research. A risk of bias assessment was performed for each included study using the Joanna Briggs Institute (JBI) checklist, ensuring all selected studies met established quality standards. Results: A total of 6 eligible research articles from a possible 1752 published until March 2024 were identified and included. We categorized the included studies into two groups based on their focus: the taxonomic composition of gut microbiota and the alpha diversity, which is the variety of organisms within a sample. Additionally, two methods were identified for assessing cognition: neuropsychological tests and physiological measurements, notably electroencephalography (EEG). The studies show varying results regarding the abundance of specific bacterial taxa and their cognitive associations. Notably, the relationship between certain bacteria and cognition may vary when analyzed at different taxonomic levels, such as phylum versus family. Conclusions: Changes in gut microbiota composition in the elderly, even without a cognitive impairment diagnosis, could potentially serve as early biological markers for Alzheimer’s disease or other dementias before mild cognitive impairment appears.

## 1. Introduction

The human aging process can be seen as a gradual and inevitable deterioration of bodily and cognitive functions. The older people get, the weaker their bodies become. The changes are biological and psychological. While the skin loses its elasticity everyday, events become harder to recall. Constipation may occur more often, as the gastrointestinal system becomes less efficient [1]. Many of those physiological and psychological changes associated with aging are increasingly understood to be implicated with gut microbiota. This is grounded in the bidirectional crosstalk between the gut and brain known as the gut–brain axis [2]. 

During the digestive process, the human gut microbiota are responsible for breaking down the nutrients in our food. Gut microorganisms transform these nutrients into metabolites, which are capable, among other things, of influencing the blood–brain barrier (BBB), that forms the walls of the brain vessels [3] and serves as a protective barrier. The progressive structural changes throughout life contribute to a decline in microorganisms capable of producing beneficial short-chain fatty acids (SCFAs). Consequently, the shortage of SCFAs results in a breakdown of the blood–brain barrier (BBB) [4]. An intact BBB allows a restricted selection of compounds to be transported between blood and brain, thus maintaining an optimal environment for the central nervous system (CNS). A broken BBB is permeable to toxins, pathogens, immune cells, or molecules, whilst blocking the transmission of beneficial nutrients. Thus, with age, the CNS loses its homeostatic function as a result of age-related gut microbiota changes [5]. 

The human gut microbiota are a community of microorganisms that inhabit the intestines. Li and colleagues [6] review a gut microbiota evolution, where the sterile at-birth gut microbiota are rapidly colonized by the mother’s skin (cesarean section) or vaginal (vaginal delivery) bacteria. During the first three years, the gut microbiota composition is dependent on the feeding practice, whether it is breast milk, formula milk, or solid food. By reaching adulthood, the gut microbiota composition becomes fully formed. In healthy adults, the gut microbiota remain stable, with Firmicutes and Bacteroidetes being the dominant phyla that harbor the gut, making up 90% of the population [7]. The healthy adult gut microbiota are also abundant in Actinobacteria, Fusobacteria, Proteobacteria, and Verrucomicrobia. Yet, the actual composition of the gut microbiota and the ratio of the species may differ between individuals. The microbiota variability may be driven by, amongst many factors, the diet [8,9], geography [10], physical activity [11], or stress level [12]. 

We can add the aging process as another factor, which may negatively affect gut microbiota taxonomic composition. The healthy elderly population’s gut microbiota are characterized by a lower abundance of Bifidobacterium and Lactobacillus, which represent the beneficial bacteria genera, and an increased presence of pro-inflammatory microbes, namely, Enterobacteriaceae and Clostridia [13]. Healthy young adults and midlife individuals’ gut microbiota are markedly richer compared to healthy elderly individuals’ microbiota composition [14]. These changes are found to be associated with gastrointestinal diseases, dysfunctional internal organs, lower caloric needs, chewing and dental problems, reduced physical activity, and reduced immunity [15]. Moreover, there is growing evidence that gut microbiota can affect the brain and behavior. Age-related gut microbiota dysbiosis, evident in the elderly population, is argued to be associated with neuronal degeneration and dysfunction [16].

Research shows that the gut microbiota of individuals with cognitive disorders differ from those of neurologically healthy elderly populations. Cognitive functions tend to decline with age, particularly memory. Episodic, working, and recognition memory are the most susceptible to age-related decline [17]. One of the most common age-related cognitive impairments is dementia [18], and Alzheimer’s disease (AD) is the most severe type of dementia.

Studies investigating the gut microbiota in individuals with AD have consistently reported an increased abundance of pro-inflammatory phyla and a decrease in anti-inflammatory phyla when compared to age-matched control groups. For instance, Vogt et al. [19] observed a notable decrease in the Firmicutes phylum, an elevation in the pro-inflammatory Bacteroidetes phylum, and a decrease in the Actinobacteria phylum. This shift in the latter phyla was primarily driven by a significant reduction in the Bifidobacterium genus, amongst AD participants in the United States, known for its anti-inflammatory properties. Additionally, Vogt et al.‘s study of AD patients and dementia participants elsewhere [20] found that these individuals exhibited reduced diversity in their gut microbiota when compared to normally aging control groups. Similarly, Cattaneo et al. [21] discovered that the gut microbiota of Italian AD participants displayed a higher prevalence of the inflammatory-enhancing Escherichia/Shigella genus and a lower abundance of the inflammatory-decreasing E. rectale species. These collective findings highlight the consistent alterations in gut microbiota composition amongst individuals with AD, suggesting potential implications for the inflammatory status and overall gut health in the context of the disease. 

However, the aging process does not always result in deterioration that interferes with daily living. A person’s experience of aging varies from one person to another. Even as individuals grow older, they can maintain a high level of cognitive and physical function, free from diseases and disabilities [22]. While there is a growing body of literature exploring the link between human gut microbiota and cognitive functions in cognitively impaired elderly individuals, the impact of gut microbiota on cognitive functioning in the healthy elderly population has been largely overlooked.

Although Freedman et al. [23] reported that 10% of adults aged 70 and older were diagnosed with dementia in 2019, the research focus on the remaining 90% of the aging population, who do not have dementia, is surprisingly limited. This gap is notable, especially considering the incurable nature of Alzheimer’s disease (AD). It is essential to explore how aging affects those free from AD to understand if they experience ‘normal’ aging and whether their cognitive functioning can be enhanced. In the current literature, such as the reviews by Białecka-Dębek et al. [24] and others, there is a noticeable lack of emphasis on the healthy aging population in the context of gut microbiota and cognitive functioning. Furthermore, when this population is considered, as seen in studies by Badal et al. [25] and Ticinesi, Tana, and Nouvenne [26], the number of studies included is often limited, highlighting the need for more comprehensive research in this area.

To our knowledge, this systematic review is the first to explore the correlation between microbiota composition and cognitive functions in a normally aging human population. We will use the term ‘Intact’ to describe individuals who scored higher on cognitive tests compared to the population’s average, and ‘Impaired’ for those with relatively lower scores. It is important to note that both Intact and Impaired groups are considered cognitively healthy. This review investigates whether there are differences in gut microbiota among older adults who are cognitively healthy, depending on their cognitive functioning levels. Our literature review addresses two primary questions: (1) Are there noticeable differences in gut microbiota composition between the Intact and Impaired healthy elderly? (2) Do these differences follow a consistent pattern across the studies reviewed? We will also examine the similarities in gut microbiota profiles between Impaired older adults and those with neurodegenerative diseases, and explore potential mechanisms linking cognitive impairment with gut microbiota changes. This review proposes the hypothesis that gut microbiota composition alterations could serve as potential early biological markers for Alzheimer’s disease, identifiable even before the mild cognitive impairment onset.

## 2. Materials and Methods

### 2.1. Search Strategy

PRISMA guidelines were followed in conducting this systematic literature search [27]. In the identification process, the Medline (PubMed), EBSCO, Web of Science, and Scopus (Full search strategies for electronic databases (Table S1) and search term history (Table S2) for each electronic database are provided in the Supplementary Materials) electronic bibliographic databases were used. The initial searches were carried out by three of the authors in December 2022, followed by a rigorous process of critical reading and synthesis of the results. Consequently, the searches were comprehensively updated in March 2024 to reflect the latest developments and insights in the field. Where there was disagreement, a fourth author made the final decision. The search strategy used the following keywords: (“human*” OR “people” OR “individual*” OR “healthy” OR “neurologically healthy” AND (“older adults” OR “aging” OR “older” OR “senior*” OR “old” OR “elderly” OR “oldest”) AND (“gut microbiota*” OR “gut-microbiome” OR “gut microbiome composition” OR “gut microbiota composition” OR “gut-microbiome” OR “microbiome” OR “microbial diversity” OR “gut microbiome diversity” OR “gut microflora” OR “gut-brain axis” OR “dysbiosis” OR “gut bacteria” OR “gastrointestinal microbiota”) AND (“cognitive flexibility” OR “cognitive decline” OR “cognitive” OR “cognitive health” OR “cognition” OR “cognitive function*” OR “cognitive performance”). No limits to the search strategy were applied.

### 2.2. Selection Process and Risk of Bias

**Figure 1:**
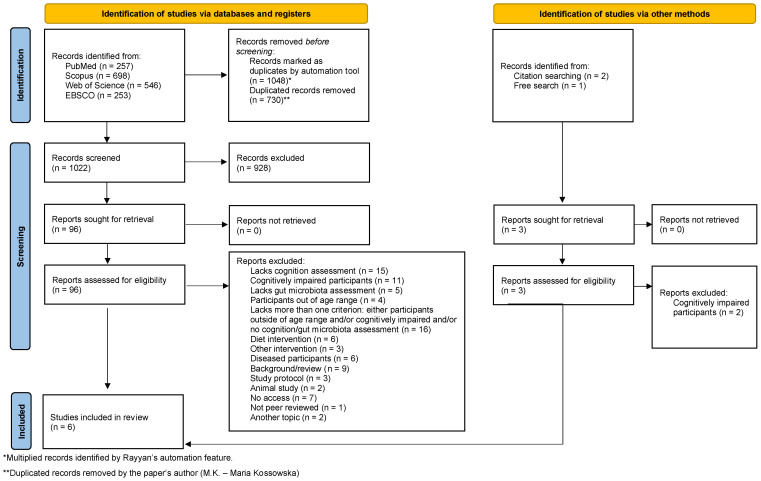
PRISMA flow diagram.

Rayyan software [28] was used for the synthesis and collation of data. In the process of literature screening, each query was checked, firstly, by the title, abstract, and keywords, to exclude articles that did not appear to be relevant within the scope of this review. Three authors (M.K., S.O., and W.M.) independently screened and retrieved each record. The inclusion criteria were (1) peer-reviewed journals, (2) a neurologically, cognitively, and medically healthy population, (3) older adults (due to the limited records, the older adults were identified as over 45 years of age), (4) gut microbiota analysis, and (5) cognitive function assessment. Following that, the full text was analyzed to assess eligibility. The exclusion criteria were (1) wrong publication type, (2) wrong sample population, (3) wrong study design, (4) lacking one or more of the inclusion criteria, and (5) animal study. Each outcome domain was sought for all measures, time points, and analyses. Detailed exclusion criteria, with an exact number of excluded records, are described in Figure 1.

A risk of bias assessment was conducted for each of the included studies using the Joanna Briggs Institute (JBI) checklist [29], which is widely recognized for assessing cross-sectional study quality. The checklist consists of five domains: selection, exposure, outcome, confounding, and analysis. The quality assessment tool comprised eight items, and specific criteria were set in advance to determine if each criterion was met. Criteria meeting the quality standard were assigned a “yes” answer, while those not meeting the standard were assigned a “no” answer, or marked as “unclear” if some information was missing. Since the item “3. Was the exposure measured in a valid and reliable way?” was not applicable across all included studies, it was removed from the final assessment. Studies that scored four or more “yes” answers were considered of high quality, while those scoring fewer than four were considered of low quality. Publications derived from pre-existing cohorts were evaluated based on the information available in the original publications. Three authors (M.K. (Maria Kossowska), A.B., and M.K. (Marcelina Karbowiak)) independently assessed and scored the included studies. To evaluate the agreement between the three authors’ scores, Kendall’s W test (w = 0.79, *p* < 0.001) was performed. Table 1 below summarizes the assessment of bias of the included studies. Risk of bias assessment for the included studies, conducted by three authors, is provided in the Supplementary Materials (Table S3).

## 3. Results

### 3.1. Study Selection

The process of literature search is presented in Figure 1. All identified records were exported to Rayyan software [28] for further screening (n = 1752). Multiplied records identified by Rayyan’s automation feature (n = 1048) were manually analyzed before deletion to avoid the risk of missing studies or misclassification. A total of 730 duplicate records were removed. Of the remaining 1022 articles, 6 studies were identified that met the inclusion criteria for this review. Three studies initially appeared to meet the inclusion criteria but were ultimately excluded. Two of them investigated the relationship between gut microbiota and cognition in elderly individuals without measuring their cognitive health, nor did they directly state that the subjects were healthy [36,37]. Likewise, the third study failed to evaluate cognitive health or verify the health status of its participants, with the subjects’ ages falling outside the specified range [38].

### 3.2. Study Characteristics 

The selected citations, summarized in Table 2, are cross-sectional studies published between 2017 and 2022, originating from the United States, the United Kingdom, Australia, and Israel. The mean age of participants ranged between 63 and 74 years, with two studies including textbook cases of older adults aged 65 years and above [39]. The remaining four studies included participants aged 40 years and above. Five of the six papers included explicitly described the characteristics of the study participants.

Manderino et al. [34] and Anderson et al. [30] used the same population, recruited from a local community recreation and wellness center, which comprised individuals without any medical conditions and did not constitute a clinical population. Haimov et al. [32] included insomniacs from a community center, but they were not part of any clinical population. In the other three articles, participants were derived from existing cohorts recruited for other study purposes, such as the TwinsUK British twin cohort [35], the Australian Research Council Longevity Intervention (ARCLI) [33], and an unspecified longitudinal study conducted in the southeastern United States [31]. Canipe et al. [31] and Komanduri et al. [33] reported that their participants underwent assessments for medical conditions.

Moreover, all included citations consistently declared their participants as “healthy” or “cognitively healthy”. While five studies assessed participants’ cognitive status using tools such as the Mini-Mental State Examination (MMSE) or Montreal Cognitive Assessment (MoCA), one study [30] did not specify the screening tool employed. However, it can be inferred that Canipe et al. [31] included participants in their study who exhibited signs of cognitive impairment, as indicated by the mean MoCA score of 26.21 ± 4.16. This suggests that individuals with scores as low as 22.05 were included in the study. However, previous studies, such as Damian et al. [40], have established a cutoff score of 24 as an optimal diagnostic threshold for elderly populations. This finding does not align with Canipe et al.’s participant selection. On the other hand, Luis et al. [41] proposed a threshold of 23 and below, which supports participant selection in Canipe et al.’s study. The appropriateness of participant selection in the study is debatable and raises questions regarding its validity.

To evaluate cognitive functioning in older adults, assessments were conducted using measurements known for their sensitivity to age-related cognitive changes commonly observed in the elderly, including executive function, memory, processing speed, and language abilities. All cited references employed neuropsychological tests to measure cognitive abilities. In the case of Canipe et al. [31], the authors investigated the influence of gut microbiota on cognitive functions and their psychophysiological correlates. In this study, event-related potentials (ERPs), an electrophysiological brain imaging method, were used. 

In all studies, gut bacterial taxa were analyzed using the 16S rRNA method, with the majority investigating the taxonomic composition, while three studies specifically examined its diversity [31,33,35]. Although one study limited the microbiota analysis to the phylum level of bacteria [34], the remaining studies also investigated family and order levels.

### 3.3. Heterogeneity 

Despite the shared objective of investigating the relationship between specific bacterial taxa and cognition in healthy adults, there is notable heterogeneity among the included studies. While all studies involve individuals declared cognitively healthy, they can be categorized as either middle-aged or older adults. The broad age range, from 40 to 89 years old, may introduce uncertainties regarding the potential influence on the outcomes of these studies. Moreover, there is a wide range of neuropsychological tasks employed to assess cognitive abilities across the studies.

Furthermore, the reported outcomes reveal correlations between cognitive abilities and bacteria at different classification levels. This heterogeneity poses challenges in identifying a precise gut microbiota composition that predicts specific cognitive functioning. Consequently, this review aims to adopt a systematic narrative approach, considering the heterogeneity in terms of population characteristics, neuropsychological tasks, and outcomes of microbiota analysis.

## 4. Findings

The studies included in this analysis can be classified into two groups based on their focus on the association between gut microbiota composition and cognition in older adults (Table 3). The first group consists of studies that examined the taxonomic compositions of the gut microbiota, while the second group investigated alpha diversity, which refers to the within-sample diversity of organisms.

Regarding the assessment of cognition, two main approaches were also identified. The first approach involved the use of neuropsychological tests to evaluate cognitive function. The second approach involved the application of physiological measurements, specifically electroencephalography (EEG), to assess cognitive functioning (see Table 3 for details).

### 4.1. Microbiota Composition and Behavioral Tests

The analysis of the intricate relationship between gut microbiota composition and cognitive abilities revealed a variety of patterns among bacterial species. Specifically, a higher presence of Verrucomicrobia was linked to improved verbal memory, visual scanning, and working memory [34]. Additionally, two different studies found that an increased abundance of Verrucomicrobia was positively associated with cognitive flexibility [30,34].

In contrast, Firmicutes exhibited a diverse influence, with increased levels linked to better immediate and delayed recall [34]. Delving deeper into specific family levels, a higher presence of Gemellaceae was associated with improved concentration and memory speed, mirroring a similar trend observed with Clostridiaceae, which was linked to enhanced attention continuity and working memory quality [33]. Yet, a lower abundance of the Ruminococcus gauvreauii group correlated with worsened spatial working memory [32]. Furthermore, an increased abundance of Carnobacteriaceae was linked to improved episodic secondary memory quality, while the presence of Blautia correlated with a lengthened reaction time. Up to this point, Firmicutes appear to be beneficial bacteria [32]. However, within the same study cohort, it was found that an increased presence of Lachnospiraceae correlated with more errors in the spatial working memory task [32]. Conversely, lower levels were associated with a longer reaction time, emphasizing the intricate and multifaceted impact of Firmicutes.

Actinobacteria exhibited a consistent role, as both lower levels of the family Propionibacteriaceae were linked to worsened spatial working memory [32], and higher levels of the family Micrococcaceae correlated with improved memorization speed [33].

Bacteroidetes displayed somewhat mixed effects across different levels of classification. An increase in the phylum Bacteroidetes was associated with poorer immediate and delayed recall [34]. However, at the family level, higher levels of Bacteroidaceae, Barnesiellaceae, and Rikenellaceae were linked to improved concentration, faster memory speed, and better attention continuity [33]. Yet, a lower level of Tannerellaceae correlated with worsened spatial working memory [32].

Proteobacteria presented contrasting effects, where a higher abundance of this phylum and the family Alcaligenaceae correlated with poorer scores in verbal learning and a decreased quality of working memory, respectively [33]. Additionally, a lower level of the order Burkholderiales and class Betaproteobacteria correlated with a longer reaction time [35]. These findings emphasize the nuanced relationships between specific microbial taxa and cognitive functions, providing insights into the intricate interplay within the gut–brain axis.

### 4.2. Alpha Diversity and Behavioural Tests

Two studies indicated that behavioral measures are linked to the level of alpha diversity in the gut microbiome—showing poorer performance in paired-associate learning, spatial working memory [31], and lower gut-microbiome diversity associated with verbal fluency and longer reaction time [35]. The third study [33] did not observe significant associations between alpha diversity and cognition in older adults.

### 4.3. Alpha Diversity and Physiological Measurements

Several electrophysiological measures demonstrated significant associations with microbiome diversity, complementing the links observed with behavioral measures [31]. Regarding the connection between the alpha diversity of the microbiome and N1 amplitude (more negative), individuals with higher amplitudes exhibited greater microbiome diversity. A higher peak amplitude of the N1 corresponded to better cognitive function, suggesting that increased microbial diversity in the gut is linked to enhanced attentional function.

Furthermore, a decreased alpha diversity of the gut microbiome predicted an increase in N2 latency to peak amplitude. Shorter latency indicates better cognitive function, and in this context, microbial diversity in the gut is associated with sustained attention. Again, the greater the alpha diversity, the better the sustained attention.

Greater P3 amplitude was related to a less diverse gut microbiome. Individuals with cognitive decline might exhibit an increase in the latency and amplitude of the P3 component, but greater alpha diversity corresponds to improved decision-making processes.

## 5. Discussion

One of the most surprising discoveries in the field of neuroscience’s microbiome research is the recognition that gut microbiota can influence the brain and behavior [42]. The gut–brain axis is a fascinating frontier, especially in the context of neurodegenerative diseases, particularly dementia. Increasingly, research is concentrating on the potential roles of gut microbiota composition and their metabolites, either as biomarkers for cognitive health or as interventions in populations at risk. Inflammation as a potential mechanism explaining probiotic influence on the brain and cognition has garnered significant attention in the scientific community. Evidence suggests that microbiota-related indicators of inflammatory processes in the body could serve as biomarkers of depression. A study [43] examining Major Depressive Disorder (MDD) patients found significant differences in the gut microbiota of MDD patients, along with elevated levels of inflammation-related biomarkers. Researchers identified that the Lachnospiraceae family within the Firmicutes phylum demonstrated significant correlations with differential inflammation-related factors and the Hamilton Depression Rating Scale (HDRS) scores. This suggests that alterations in gut microbiota might lead to changes in systemic inflammation markers, which correlate with the severity of depression. These insights support the hypothesis that imbalances in gut microbiota contribute to systemic inflammation, potentially impacting mental health and brain function. In this article, we focused on the relationship between gut microbiota and cognitive functioning in the healthy elderly population. The findings summarized here showed gut microbiota’s nuanced and multifaceted effects on different cognitive functions, illustrating the complex interactions within the gut–brain axis. One of the most promising conclusions is the possibility of using gut microbiota composition information and their metabolites as biomarkers of the cognitive state or neurodegenerative disease of the host.

Although some researchers (e.g., [26]) argue that fecal microbiota biodiversity indices may not serve as reliable biomarkers of cognitive aging, other research shows promising results. For example, the findings of Canipe et al. [31] suggest that higher alpha diversity might be an indicator of better cognitive functions in a healthy population. Studies exploring the link between microbiome and dementia have identified several abnormalities in microbiome composition that may serve as biomarkers of present or future problems with cognition [44]. One of them, identified also in our review as a protective factor for cognitive health in the healthy aged population, is microbiota biodiversity. We found that in most studies, a higher microbial diversity in the gut is related to better cognitive functioning or its physiological indicators. Only two studies found no correlation between those variables, and no studies reported a negative relationship (i.e., poorer cognitive performance accompanied by a higher diversity of gut microbiota).

Other studies point to specific species of gut bacteria being related to better cognitive functioning. For example, the abundance of bacteria from the phylum Verrucomicrobia was found to be positively associated with verbal memory, visual scanning, working memory, and cognitive flexibility [30,34], and the phylum Firmicutes was associated with—among others—better immediate and delayed recall. Specific families within Firmicutes, such as Gemellaceae and Clostridiaceae, were associated with improved concentration, memory speed, attention, and working memory quality. The Ruminococcus gauvreauii group and Carnobacteriaceae within this phylum also showed positive correlations with certain cognitive functions, while Lachnospiraceae had a reverse association with cognition, e.g., spatial working memory [32,33,34]. It seems that the Firmicutes/Bacteroidetes (F/B or Bacillota-to-Bacteroidota, according to the newest nomenclature, [45]) ratio is another important factor in cognitive functioning prediction. The F/B ratio is often studied in the context of human health, particularly concerning obesity and other metabolic conditions, but it seems that it is also connected to cognitive health. In general, any deviation from F/B is considered dysbiosis and harms the host. The F/B ratio is recognized as an important index of gut microbiota health and is also influenced by the amount of physical exercise (e.g., [46]), so it is not surprising that it can also influence the cognitive status of the host. It seems that higher amounts of Bacteroidota are related to better cognition [32,33], but some studies described in this review showed the opposite pattern [34]. Still, most studies on this topic were conducted on people suffering from different forms of neurodegeneration, so we cannot draw any conclusions about a healthy aging population.

Several other environmental and host-related factors may be involved in mediating the possible link between gut microbiota and cognition. For example, recent studies showed unexpected results in different countries. Recent investigations have demonstrated an increase in beneficial Bifidobacterium taxa among Chinese individuals with AD [47] and cognitively impaired participants [48]. Similarly, Bifidobacterium and Lactobacillus were found to be elevated in Japanese participants with dementia [49]. Bairamian et al. [50] suggest that these variations in gut microbiota composition among Chinese individuals with AD may be influenced by geographical factors and diverse dietary patterns. Białecka-Dębek et al. [24] attribute the surprising outcomes to methodological issues. They argue that the seemingly insignificant higher abundance of anti-inflammatory Lactobacillus and Bifidobacterium in the studies conducted by Saji et al. [49] and Lu et al. [48] could be attributed to the inclusion of participants with mild cognitive impairment in the non-dementia control group, as determined by the reported Mini-Mental State Examination (MMSE) scores [51]. These contrasting findings highlight the complex nature of gut microbiota research when it comes to AD, and underscore the need for a careful consideration of various factors such as geographical location, diet, and methodological rigor in future investigations.

The reviewed studies are primarily observational, featuring a cross-sectional design. This methodology poses a challenge in establishing causation between gut microbiota composition and cognitive outcomes due to the potential influence of uncontrolled covariates. The studies reviewed have varied in their approach to addressing these complexities. It has been proposed that microbiota can be shaped by host genetics, diet, age, the mode of birth, antibiotics, obesity, diabetes, allergies, hypertension, autoimmune disorders, cardiovascular diseases, and bowel diseases [52]. While Manderino et al.’s [34] study did not adjust for specific covariates, suggesting a gap in controlling for factors known to influence microbiota, other studies have made concerted efforts to account for these variables. Anderson et al.’s [30] study controlled for hypertension and carbohydrate intake, acknowledging the role of diet. Similarly, Haimov et al.’s [32] inclusion of lifestyle and medical conditions represents a more robust attempt to isolate the effects of sleep quality and cognitive performance on gut microbiota composition. Verdi et al.’s [35] study further illustrates the complexity of accounting for covariates like physical frailty, medication use, and diet, suggesting that factors beyond these may also significantly impact gut microbiota composition. Canipe et al.’s [31] study, which included education as a significant covariate, further emphasizes the importance of considering demographic factors in understanding the gut–brain axis. Thus, Komanduri et al.’s [33] regression analyses were adjusted for demographic variables, including age, sex, and BMI, to evaluate the combined contribution of significant bacterial families in predicting cognition. This limitation is crucial in establishing whether gut microbiota can serve as a biomarker for cognitive status.

Building on this premise, a future longitudinal study is warranted to investigate the association between the gut microbiota of healthy individuals and those with Alzheimer’s disease (AD). The objective of this study is to assess whether healthy older adults with microbiota like those found in AD patients are more susceptible to developing neurodegenerative diseases, and whether there are similarities between these two populations. By monitoring the microbiota composition of the subjects over an extended period, we may be able to identify potential alterations in gut microbiota composition that could serve as a potential biological marker for the early diagnosis of Alzheimer’s disease before the onset of mild cognitive impairment.

The outcomes of the reviewed studies should be approached with caution due to an additional consideration. There is an observable deficiency in methodological rigor concerning the management of multiple comparisons. Although some studies [31,35] explicitly employ statistical corrections to mitigate the risk associated with multiple comparisons, others [30,32,33,34] refrain from adjusting for these comparisons due to their exploratory nature. This underscores the complexity of interpreting the impact of the gut–brain axis on cognition and necessitates careful consideration when drawing conclusions from these data. To enhance our understanding of the gut–brain axis’s influence on cognition, future meta-analyses could address these issues by applying appropriate corrections across pooled data. Despite these challenges, the field exploring the relationship between the gut–brain axis and cognitive function is rapidly evolving, signaling a clear need for further studies to unravel the complex interactions between gut microbiota and the brain.

## Figures and Tables

**Table 1:** Risk of bias assessment of the included studies.

		Citation [Reference]					
Item No	JBI Item	Anderson et al., 2017 [30]	Canipe et al., 2021 [31]	Haimov et al., 2022 [32]	Komanduri et al., 2021 [33]	Manderino et al., 2017 [34]	Verdi et al., 2018 [35]
1.	Were the criteria for inclusion in the sample clearly defined?	Yes	Yes	Yes	Yes	Yes	Yes
2.	Were the study subjects and the setting described in detail?	Unclear	Yes	Yes	Yes	Yes	Yes
3.	Was the exposure measured in a valid and reliable way?	Not Applicable	Not Applicable	Not Applicable	Not Applicable	Not Applicable	Not Applicable
4.	Were objective, standard criteria used for measurement of the condition?	Yes	Yes	Yes	Yes	Yes	Unclear
5.	Were confounding factors identified?	Yes	Yes	Yes	Yes	Yes	Yes
6.	Were strategies to deal with confounding factors stated?	Yes	Yes	Yes	Yes	Yes	Yes
7.	Were the outcomes measured in a valid and reliable way?	Yes	Yes	Yes	Yes	Yes	Yes
8.	Was appropriate statistical analysis used?	Yes	Yes	Yes	Yes	Yes	Yes
	Overall Appraisal	Include	Include	Include	Include	Include	Include

**Table 2:** Overview of characteristics of reviewed studies.

Country [Reference]	N, % Sex, Nationality	Age	Cognitive Function Assessment (Score)	Cognitive Test	Microbiome Assessment
USA [30]	37, 73% female	64.59 ± 7.54	Stroop Word (48.51 ± 6.71) Stroop Color (48.30 ± 6.87) Stroop Color-Word subset (51.22 ± 10.22)	Stroop Word, Stroop Color, Stroop-Color-Word subset	Fecal samples, bacterial 16S rRNA
Southeastern US [31]	63, 43.27% male	74.63 ± 4.26	MoCA (26.21 ± 4.16)	ERP active discrimination, ERP passive oddball, CANTAB	Fecal samples, bacterial 16S rRNA
Israel [32]	72, 77.77% female	73.19 ± 5.73	MMSE (>26)	CANTAB	Fecal samples, bacterial 16S rRNA
Australia [33]	69, 49% male	65.06 ± 4.01	MMSE (28.78 ± 1.29)	QESM, QWM, PoC, CoA, SoM	Fecal samples, bacterial 16S rRNA
USA [34]	43, Intact 32% female, Impaired 33.3% female	Intact 64.08 ± 6.49, Impaired 64.06 ± 9.37	MMSE (Intact 29.28 ± 0.98, Impaired 28.00 ± 1.85)	FAB, TMT-A, TMT-B, SCWT, HVLT-R, ROCF, verbal fluency, animal naming	Fecal samples, bacterial 16S rRNA
UK [35]	1551, 90% female	63 (40–89)	MMSE (mean 29)	verbal fluency, DLRT, CANTAB-PAL	Fecal samples, bacterial 16S rRNA

Abbreviations: USA, United States of America; Southeastern US, southeastern United States; UK, United Kingdom; CANTAB, Cambridge Neuropsychological Test Automated Battery; CANTAB-PAL, Cambridge Neuropsychological Test Automated Battery–Paired Associates Learning; CoA, Continuity of Attention; DLRT, Deary–Liewald Reaction Time; ERP, Event-related potential, FAB, The Frontal Assessment Battey; HVLT-R, Hopkins Verbal Learning Test-Revised; MMSE, Mini-Mental State Examination; MoCA, Montreal Cognitive Assessment; PoC, Power of Concentration; QESM, Quality of Episodic Secondary Memory; QWM, Quality of Working Memory; ROCF, Rey–Osterrieth Complex Figure task; SCWT, Stroop Color Word Test; SoM, Speed of Memory; TMT-A, Trail Making Test A; TMT-B, Trail Making Test B; 16S rRNA, 16S ribosomal RNA.

**Table 3:** Summary of outcomes from reviewed studies.

Country [Reference]	Taxonomic Composition/Diversity Pattern	Cognitive Functions/Psychophysiological Measures
*Microbiota composition/Alpha diversity and behavioral tests*		
USA Anderson et al., 2017 [30]	↑ Verrucomicrobia ↑ Verrucomicrobia	↑ Stroop Word
		↑ Stroop Color
	↑ Lentisphaerae	↑ Stroop Color-Word subset
Israel Haimov et al., 2022 [32]	↑ Lachnospiraceae (Firmicutes)	↑ SWM (less SWMBE—Spatial Working Memory Between Errors)
	↓ Ruminococcus gauvreauii group (Firmicutes)	
	↓ Propionibacteriaceae (Actinobacteria)	
	↓ Tannerellaceae (Bacteroidetes)	
	↓ Blautia (Firmicutes)	↑ MTTLMD (Median Reaction Latency)
	↓ Lachnospiraceae (Firmicutes)	
Australia Komanduri et al., 2021 [33]	↑ Carnobacteriaceae (Firmicutes)	↑ QESM
	↑ Clostridiaceae (Firmicutes)	↑ QWM
	↑ Alcaligenacea (Proteobacteria)	↓ QWM
	↑ Bacteroidaceae, (Bacteroidetes)	↑ PoC
	↑ Barnesiellaceae (Bacteroidetes)	
	↑ Gemellaceae (Firmicutes)	
	↑ Rikenellaceae (Bacteroidetes)	
	↑ Clostridiaceae (Firmicutes)	↑ CoA
	↑ Rikenellaceae (Bacteroidetes)	
	↑ Verrucomicrobia	↓ CoA
	↑ Bacteroidaceae (Bacteroidetes)	↑ SoM
	↑ Barnesiellaceae (Bacteroidetes)	
	↑ Gemellaceae (Firmicutes)	
	↑ Micrococcaceae (Actinobacteria)	
USA Manderino et al., 2017 [34]	↑ Verrucomicrobia	↑ TMT-A
		↑ TMT-B
		↑ SCWT Word
		↑ SCWT Color
		↑ HVLT-R Total Learning
	↑ Proterobacteria	↓ FAB
		↓ HVLT-R Recognition/Discrimination
		↓ FAS
	↑ Firmicutes	↑ CFT Immediate and delayed recall
	↑ Baceroidetes	↓ CFT Immediate and delayed recall
UK Verdi et al., 2018 [35]	↑ alpha diversity	↑ verbal fluency
	↓ alpha diversity	↑ DLRT
	↓ order: Burkholderiales, class: Betaproteobacteria (Proteobacteria)	
*Alpha diversity and physiological measurements*		
Southeastern US Canipe et al., 2021 [31]	↑ alpha diversity	↑ N1 minimum amplitude and mean amplitude (50–190 ms; frontal clusters; target condition)
	↓ alpha diversity	↑ N2 latency to peak amplitude (200–350 ms frontal cluster; familiar condition)
		↑ P3 maximum amplitude (350–1500 ms; temporal left cluster; familiar condition)
		↑ P3 mean amplitude (350–1500 ms; temporal left cluster; familiar condition
		↑ total errors PAL
		↑ mean time success SWM

Abbreviations: CFT, Complex Figure Task; CoA, Continuity of Attention; DLRT, Deary–Liewald Reaction Time; FAB, The Frontal Assessment Battey; HVLT-R, Hopkins Verbal Learning Test-Revised; MTTLMD, Multitasking Test of Median Reaction Latency; N1, negative event-related potential (peak between 80 and 120 ms after the onset of a stimulus); N2, negative event-related potential (peak between 200 and 300 ms after the onset of a stimulus); P3 (peak between 300 and 600 ms after the onset of a stimulus); PAL, Paired Associates Learning; PoC, Power of Concentration; QESM, Quality of Episodic Secondary Memory; QWM, Quality of Working Memory; SCWT, Stroop Color Word Test; SoM, Speed of Memory; SWM, Spatial Working Memory; SWMBE, Spatial Working Memory Between Errors; TMT-A, Trail Making Test A; TMT-B, Trail Making Test B.

## Data Availability

The raw data supporting the conclusions of this article will be made available by the authors on request.

## Supplementary Materials

The following supporting information can be downloaded at: https://www.mdpi.com/article/10.3390/nu16060852/s1, Table S1: Full search strategies for electronic databases; Table S2: Search term history, Table S3: Risk of bias assessment for the included studies, conducted by three authors.
