# Supplementary material for: The Interplay between Gut Microbiota and Cognitive Functioning in the Healthy Aging Population: A Systematic Review
Source: Nutrients. 2024 Mar 15;16(6):852. doi: 10.3390/nu16060852 (PMC10974508; doi:10.3390/nu16060852)
Supplement: Supplementary file 1 [file nutrients-16-00852-s001.zip › nutrients-2892981-supplementary.pdf]

Supplementary Material

Table S1. Full search strategies for electronic databases

	Concept 1	Concept 2	Concept 3	Concept 4
**Key Concepts**	**Healthy human**	**Older adult**	**Gut microbiota**	**Cognitive functions**
Vocabulary Terms	"human" OR "people" OR "individual" OR "healthy" OR "neurologically healthy"	"older adults OR "aging" OR "older" OR "senior*" OR "old" OR "elderly" OR "oldest"	"gut microbiota" OR "gut-microbiome" OR "gut microbiome composition" OR" gut microbiota composition" OR "gut-microbiome" OR "microbiome" OR "microbial diversity" OR "gut microbiome diversity" OR "gut microflora" OR "gut brain axis" OR "dysbiosis" OR "gut bacteria" OR "gastrointestinal microbiota"	"cognitive flexibility" OR "cognitive decline" OR "cognitive" OR "cognitive health" OR "cognition" OR "cognitive function" OR "cognitive performance"

Each concept will be joined together using AND as a search strategy for all databases.

Table S2. Search terms history

Electronic Bibliographic Databases	Query	Filters	Results
PubMed	("human*"[Title/Abstract] OR "people"[Title/Abstract] OR "individual*"[Title/Abstract] OR "healthy"[Title/Abstract] OR "neurologically healthy"[Title/Abstract]) AND ("older adults"[Title/Abstract] OR "aging"[Title/Abstract] OR "older"[Title/Abstract] OR "senior*"[Title/Abstract] OR "old"[Title/Abstract] OR "elderly"[Title/Abstract] OR "oldest"[Title/Abstract]) AND ("gut microbiota*"[Title/Abstract] OR "gut-microbiome"[Title/Abstract] OR "gut microbiome composition"[Title/Abstract] OR "gut microbiota composition"[Title/Abstract] OR "gut-microbiome"[Title/Abstract] OR "microbiome"[Title/Abstract] OR "microbial diversity"[Title/Abstract] OR "gut microbiome diversity"[Title/Abstract] OR "gut microflora"[Title/Abstract] OR "gut brain axis"[Title/Abstract] OR "dysbiosis"[Title/Abstract] OR "gut bacteria"[Title/Abstract] OR "gastrointestinal microbiota"[Title/Abstract]) AND ("cognitive flexibility"[Title/Abstract] OR "cognitive decline"[Title/Abstract] OR "cognitive"[Title/Abstract] OR "cognitive health"[Title/Abstract] OR "cognition"[Title/Abstract] OR "cognitive function*"[Title/Abstract] OR "cognitive performance"[Title/Abstract])	None	171
EBSCO	AB (((AB human*) OR (AB people) OR (individual*) OR (healthy) OR (neurologically healthy))) AND AB (((older adults) OR (aging) OR (old) OR (older) OR (oldest) OR (senior*) OR (elderly))) AND AB (((gut microbiota*) OR (gut-microbiome) OR (gut microbiome composition) OR (gut microbiota composition) O (microbiome) OR (microbial diversity) OR (Gut microbiome diversity) OR (gut microflora) OR (gut-brain axis) OR (dysbiosis) OR (gastrointestinal microbiota))) AND AB (((cognitive flexibility) OR (cognitive decline) OR (cognitive) OR (cognitive health) OR (cognition) OR (cognitive function*) OR (cognitive performance)))	None	139
Web of Science	(((((TS=(human*))) OR TS=(people)) OR TS=(individual*)) OR TS=(healthy)) OR TS=(neurologically healthy) AND ((((((TS=(older adults)) OR TS=(aging)) OR TS=(OLDER)) OR TS=(senior*)) OR TS=(OLD)) OR TS=(elderly)) OR TS=(OLDEST) AND ((((((((((((TS=(gut microbiota*)) OR TS=(gut microbiome)) OR TS=(gut microbiome composition)) OR TS=(gut microbiota composition)) OR TS=(gut-microbiome)) OR TS=(microbiome)) OR TS=(microbial diversity)) OR TS=(gut microbiome diversity)) OR	None	423

	TS=(gut microflora)) OR TS=(gut-brain axis)) OR TS=(dysbiosis)) OR TS=(gut bacteria)) OR TS=(gastrointestinal microbiota) AND ((((((TS=(cognitive flexibility)) OR TS=(cognitive decline)) OR TS=(cognitive)) OR TS=(cognitive health)) OR TS=(cognition)) OR TS=(cognitive function*)) OR TS=(cognitive performance)		
Scopus	((TITLE-ABS-KEY (neurologically AND healthy)) OR (TITLE-ABS-KEY (healthy)) OR (TITLE-ABS-KEY (individual*)) OR (TITLE-ABS-KEY (people)) OR (TITLE-ABS-KEY (human*))) AND ((TITLE-ABS-KEY (older AND adults)) OR (TITLE-ABS-KEY (aging)) OR (TITLE-ABS-KEY (older)) OR (TITLE-ABS-KEY (senior*)) OR (TITLE-ABS-KEY (old)) OR (TITLE-ABS-KEY (elderly)) OR (TITLE-ABS-KEY (oldest))) AND ((TITLE-ABS-KEY (gastrointestinal AND microbiota)) OR (TITLE-ABS-KEY (gut AND bacteria)) OR (TITLE-ABS-KEY (dysbiosis)) OR (TITLE-ABS-KEY (gut-brain AND axis)) OR (TITLE-ABS-KEY (gut AND microflora)) OR (TITLE-ABS-KEY (gut AND microbiome AND diversity)) OR (TITLE-ABS-KEY (microbial AND diversity)) OR (TITLE-ABS-KEY (microbiome)) OR (TITLE-ABS-KEY (gut-microbiome)) OR (TITLE-ABS-KEY (gut AND microbiota AND composition)) OR (TITLE-ABS-KEY (gut AND microbiome AND composition)) OR (TITLE-ABS-KEY (gut AND microbiome)) OR (TITLE-ABS-KEY (gut AND microbiota*))) AND ((TITLE-ABS-KEY (cognitive AND performance)) OR (TITLE-ABS-KEY (cognitive AND function*)) OR (TITLE-ABS-KEY (cognition)) OR (TITLE-ABS-KEY (cognitive AND health)) OR (TITLE-ABS-KEY (cognitive)) OR (TITLE-ABS-KEY (cognitive AND decline)) OR (TITLE-ABS-KEY (cognitive AND flexibility)))	None	476

Table S3.1. Risk of bias assessment of the included studies - assessed by MK (Maria Kossowska)

		Citation [Reference]					
**Item No**	**JBI Item**	**Anderson et al., 2017**	**Canipe et al., 2021**	**Haimov et al., 2022**	**Komanduri et al., 2021**	**Manderion et al., 2017**	**Verdi et al., 2018**
1.	Were the criteria for inclusion in the sample clearly defined?	Yes	Yes	Yes	Yes	Yes	Yes
2.	Were the study subjects and the setting described in detail?	Unclear	Yes	Yes	Yes	Yes	Yes
3.	Was the exposure measured in a valid and reliable way?	Not Applicable	Not Applicable	Not Applicable	Not Applicable	Not Applicable	Not Applicable
4.	Were objective, standard criteria used for measurement of the condition?	Yes	Yes	Yes	Yes	Yes	Unclear
5.	Were confounding factors identified?	Yes	Yes	Yes	Yes	Yes	Yes
6.	Were strategies to deal with confounding factors stated?	Yes	Yes	Yes	Yes	Yes	Yes
7.	Were the outcomes measured in a valid and reliable way?	Yes	Yes	Yes	Yes	Yes	Yes
8.	Was appropriate statistical analysis used?	Yes	Yes	Yes	Yes	Yes	Yes
	**Score**	6	7	7	7	7	6
	**Overall Appraisal**	Include	Include	Include	Include	Include	Include

Table S3.2. Risk of bias assessment of the included studies - assessed by AB

		Citation [Reference]					
**Item No**	**JBI Item**	**Anderson et al., 2017**	**Canipe et al., 2021**	**Haimov et al., 2022**	**Komanduri et al., 2021**	**Manderion et al., 2017**	**Verdi et al., 2018**
1.	Were the criteria for inclusion in the sample clearly defined?	Yes	Yes	Yes	Yes	Yes	Yes
2.	Were the study subjects and the setting described in detail?	Unclear	Yes	Yes	Yes	Yes	Yes
3.	Was the exposure measured in a valid and reliable way?	Not Applicable	Not Applicable	Not Applicable	Not Applicable	Not Applicable	Not Applicable
4.	Were objective, standard criteria used for measurement of the condition?	Yes	Yes	Yes	Yes	Yes	Unclear
5.	Were confounding factors identified?	Yes	Yes	Yes	Yes	Yes	Yes
6.	Were strategies to deal with confounding factors stated?	Yes	Yes	Yes	Yes	Yes	Yes
7.	Were the outcomes measured in a valid and reliable way?	Yes	Yes	Yes	Yes	Yes	Yes
8.	Was appropriate statistical analysis used?	Yes	Yes	Yes	Yes	Yes	Yes
	**Score**	6	7	7	7	7	6
	**Overall Appraisal**	Include	Include	Include	Include	Include	Include

Table S3.3. Risk of bias assessment of the included studies - assessed by MK (Marcelina Karbowiak)

		Citation [Reference]					
**Item No**	**JBI Item**	**Anderson et al., 2017**	**Canipe et al., 2021**	**Haimov et al., 2022**	**Komanduri et al., 2021**	**Manderion et al., 2017**	**Verdi et al., 2018**
1.	Were the criteria for inclusion in the sample clearly defined?	Yes	Yes	Yes	Yes	Yes	Unclear
2.	Were the study subjects and the setting described in detail?	Unclear	Yes	Unclear	Unclear	Yes	Unclear
3.	Was the exposure measured in a valid and reliable way?	Not Applicable	Not Applicable	Not Applicable	Not Applicable	Not Applicable	Not Applicable
4.	Were objective, standard criteria used for measurement of the condition?	Yes	Yes	Yes	Yes	Yes	Yes
5.	Were confounding factors identified?	Yes	Unclear	Unclear	Unclear	Unclear	Yes
6.	Were strategies to deal with confounding factors stated?	Yes	Yes	Yes	Yes	Yes	Yes
7.	Were the outcomes measured in a valid and reliable way?	Yes	Unclear	Unclear	Unclear	Unclear	Unclear
8.	Was appropriate statistical analysis used?	Yes	Yes	Yes	Yes	Yes	Yes
	**Score**	5	6	7	6	6	5
	**Overall Appraisal**	Include	Include	Include	Include	Include	Include
